# Adaptive responses and invasion: the role of plasticity and evolution in snail shell morphology

**DOI:** 10.1002/ece3.471

**Published:** 2013-01-17

**Authors:** Erica J Kistner, Mark F Dybdahl

**Affiliations:** School of Biological Sciences, Washington State UniversityP. O. Box 644236, Pullman, Washington, 99164

**Keywords:** Adaptive evolution, Canonical variate analysis, morphometric landmarks, phenotypic plasticity, Potamopyrgus antipodarum, shell morphology

## Abstract

Invasive species often exhibit either evolved or plastic adaptations in response to spatially varying environmental conditions. We investigated whether evolved or plastic adaptation was driving variation in shell morphology among invasive populations of the New Zealand mud snail (*Potamopyrgus antipodarum*) in the western United States. We found that invasive populations exhibit considerable shell shape variation and inhabit a variety of flow velocity habitats. We investigated the importance of evolution and plasticity by examining variation in shell morphological traits 1) between the parental and F_1_ generations for each population and 2) among populations of the first lab generation (F_1_) in a common garden, full-sib design using Canonical Variate Analyses (CVA). We compared the F_1_ generation to the parental lineages and found significant differences in overall shell shape indicating a plastic response. However, when examining differences among the F_1_ populations, we found that they maintained among-population shell shape differences, indicating a genetic response. The F_1_ generation exhibited a smaller shell morph more suited to the low-flow common garden environment within a single generation. Our results suggest that phenotypic plasticity in conjunction with evolution may be driving variation in shell morphology of this widespread invasive snail.

## Introduction

Only a small fraction of non-native taxa successfully establishes and becomes widespread (Mack et al. 2000), leading to a key question in invasion ecology: what characteristics of a species determine its success at invading a range of new environments? Phenotypic plasticity, which is environmentally sensitive production of alternative phenotypes by given genotypes (Stearns 1989), is widely thought to facilitate the spread of invasive species (Baker 1965; Agrawal 2001; Yeh and Price 2004; Richards et al. 2006). Adaptive and incomplete plasticity may allow invaders to survive novel environments by placing individuals within the locally optimal adaptive peak (Ghalambor et al. 2007). If invasive species exhibit or evolve greater plasticity than native species (McDowell and Lee 2002; Yeh and Price 2004), then invasives might have a fitness advantage over natives in the invaded range (Schweitzer and Larson 1999; Legar and Rice 2003). At the same time, adaptive evolution in response to local regimes of natural selection leads to genotypes specialized for different local environments, and also facilitates spread across an environmental gradient (Lee 2002; Lee and Gelembiuk 2008). There is strong evidence that adaptive evolution is the driving force behind many plant invasions (Reznick and Ghalambor 2001; Parker et al. 2003; Prentis et al. 2008) and some animal invasions (Lee et al. 2003; Kolbe et al. 2004). Hence, either evolved or plastic responses might facilitate invasion as long as plastic responses are at least partially adaptive. However, there are still few studies that examine plastic versus evolved responses in permitting the spread of invasives into new ranges (Chevin and Lande 2011).

We were interested in determining whether plasticity or evolution was driving variation among invasive populations of the freshwater snail, *Potamopyrgus antipodarum*, the New Zealand Mud Snail, in the western United States. This snail exhibits both sexual and parthenogenetic (or clonal) reproduction in its native New Zealand range, but clonal populations have invaded Europe, Australia, Japan, and the United States (Ponder 1988; Wallace 1992; Dybdahl and Kane 2005). Geographically widespread populations in Europe, the Great Lakes, and the western United States have been founded by single clonal lineages (Ponder 1988; Hauser et al. 1992; Dybdahl and Drown 2011). The pattern of these clonal invasions might suggest that phenotypic plasticity plays a role in maintaining high fitness across some environmental conditions, but studies of phenotypic plasticity in growth, survivorship, and reproduction are equivocal. Some studies suggest that invasive genotypes are broadly tolerant, while others suggest that they are opportunistic specialists (Jacobsen and Forbes 1997; Dybdahl and Kane 2005; Drown et al. 2010). On the other hand, adaptive evolution is possible in clonal populations as the accumulation of mutational variance should be rapid in invasive populations that reproduce rapidly and reach high population densities (Butin et al. 2005; Wares et al. 2005). In the western United States, invasive populations of *P. antipodarum* should have great evolutionary potential because they occupy a vast area, sometimes at high densities (Hall et al. 2003, 2006; Kerans et al. 2005).

In this article, we were particularly interested in examining the importance of evolved versus plastic responses in the shell morphology of invasive populations of *P. antipodarum* in the western United States. Snail shells are an important determinant of an aquatic snail's fitness and are known for exhibiting considerable plasticity (Kemp and Bertness 1984). Specific shell morphologies are favored under different environmental conditions such as current velocity, temperature, and predator abundance (Struhsaker 1968; Janson and Sundberg 1983; Vermeij 1995; Rolan-Alvarez et al. 1997; Bourdeau 2009). Despite this, little is no known about shell variation in the invaded range of these snails. In the western United States, invasive populations of *P. antipodarum* inhabit a wide variety of flow velocity habitats, from slow-velocity large rivers and reservoirs to high-flow rivers and streams (Dybdahl and Drown 2011). Water velocity is often an important environmental variable that favors specific shell morph adaptations (Vermeij 1995). Snails exhibiting shorter spires and larger apertures are expected to be favored in fast-velocity regimes, thereby reducing lift and drag pressure and maximizing the foot for attachment (Dussart 1987; Statzner and Holm 1989; Vermeij 1995). In their native range, *P. antipodarum* exhibits shell adaptation to water flow and these adaptations are likely the product of phenotypic plasticity. Haase (2003) found clinal variation in *P. antipodarum* shell morphology with wider snail morphs being associated with downstream sites where water flow was stronger. Our earlier work also showed that shell shape varied among populations at four geographically distinct populations along the Snake River (Idaho, U. S.) as predicted. Populations in high-velocity habitats had large apertures and small spires compared with populations in low water velocity reaches (Kistner and Dybdahl, in revision). Although there is evidence that *P. antipodarum* exhibits morphological plasticity in their native range (Negovetic and Jokela 2001; Haase 2003; Holomuzki and Biggs 2006), nothing is known about the contribution of evolved versus plastic change in explaining morphological variation among invasive populations.

In this study, we examine variation in shell morphology across a wider portion of the western U. S. range, and perform a common garden experiment to estimate the importance of plastic and evolved responses in shell morphology. We chose three populations of *P. antipodarum*: Bear River in Idaho, Polecat Creek in Wyoming, and Green River in Utah. These sites vary in average water velocity as well seasonal water velocity. As Dybdahl and Drown (2011) found little genotypic variation among these three sites using genetic markers, morphological variation may be the result of phenotypic plasticity rather than evolved specialization. In a common garden experiment, we compared shell shape between first lab generation (F_1_) and field-collected parentals for each population and among the F_1_ generations. Shell shape was analyzed using both geometric morphometric analyses and traditional shell size measures.

## Methods

### Study system

*Potamopyrgus antipodarum* is a fresh water snail native to the lakes and rivers of New Zealand. Native populations are comprised of a mixture of sexual and parthenogenetic individuals, with clonal lineages having arisen from the sympatric sexual population (Dybdahl and Lively 1995). A rich variety of clonal genotypes occur in the native range. However, invasive populations in Europe and the United States lack diversity as measured by genetic markers. A single clonal genotype, US 1, has spread rapidly in the western United States since 1987, and sometimes reaches great abundance (Hall et al. 2003, 2006; Kerans et al. 2005; Dybdahl and Drown 2011). In the native range of *P. antipodarum*, variation in shell morphology reflects adaptive responses to abiotic and biotic factors (Negovetic and Jokela 2001; Haase 2003; Holomuzki and Biggs 2006). However, little is known about how variation in shell morphology affects the success of invasive populations across broad environmental gradients.

### Study populations and collection methods

To study variation among populations in the wild and in a common garden, we collected individuals of the invasive US 1 genotype for the parental generation from three geographically distinct sites located in the western United States (Appendix A1): Polecat Creek, WY, Bear River, ID, and Green River, UT. Polecat Creek is a geothermally influenced spring-fed tributary of the Snake River (Hall et al. 2006). We collected from the population near Flagg Ranch, WY. The Bear River runs through Idaho and empties in to the Great Salt Lake, Utah. We collected from a population near the Black Canyon and Soda Springs, ID. The Green River originates in Wyoming and runs through Utah as a chief tributary of the Colorado River. We collected near Little Hole and Manila, UT. On average, the three sites differ in water flow, which is relatively stable at Polecat Creek because it is a spring-fed creek, while Bear River and Green River experience much greater water velocity with regular and seasonal flow fluctuations because they are downstream from dams. The Bear River sample site is located downstream from Grace Dam, whereas the Green River sample site is located downstream from Flaming Gorge Dam.

Adult snails, constituting the parental generation, were collected during August 2007 by sifting aquatic vegetation and substrate using wire sieves. Snails were put into plastic bags containing moist paper towels, placed in a cooler with ice, and transported to a lab at Washington State University (Pullman, WA).

### Common garden

A laboratory common garden experiment determined the level of variation in shell morphology among three distinct populations of *P. antipodarum* that is genetically based or evolved versus environmentally based or the result of plasticity.

A total of 205 snails were used in this experiment; 58 snails comprised the parental generation and 147 snails comprised the F_1_ lineage. Parental-generation snails from each of the three populations were maintained individually to initiate isofemale F_1_ lineages. The females were isolated in 148-ml plastic cups beginning on September 1, 2007. Parental snails were fed 0.24 mg of Spirulena and the water was changed on three alternating days per week. Each week, F_1_ offspring from each female were placed in a separate cup and maintained for 2 weeks, at which point they were placed individually into cups. When available, five F_1_ individuals from each female were randomly selected for the experiment. All F_1_ generation snails were fed on three alternating days per week and kept at a constant temperature of 18°C in a 12-L:12-D cycle. The water in the cups was changed on three alternating days per week. The feeding regiment increased with snail age: snails were fed 0.02 mg Spirulena until individuals reached a length of 0.8 mm, 0.04 mg when between 0.8 and 1.6 mm length, and 0.24 mg when greater than 1.6 mm length. F_1_ offspring were raised to adulthood, when they reached full size and began reproduction.

### Morphology measurement

Shells from both the parental and F_1_ generation were scrubbed clean of algae, dried, and mounted on museum gel to prevent shadows. Images were obtained using a Canon Powershot A620 digital camera on a stable stand attached to a dissecting microscope. Shells were oriented with the axis of coiling horizontal, and the aperture face up. A millimeter ruler was mounted in the plane of aperture focus. Consistent orientation of the specimen is critical to minimize random error in morphometric analysis (Schilthuizen and Haase 2010). An error series of repeated photos of the same shell were taken to quantitate orientation errors. This process was repeated until the error rate was undetectable.

**Figure 1:**
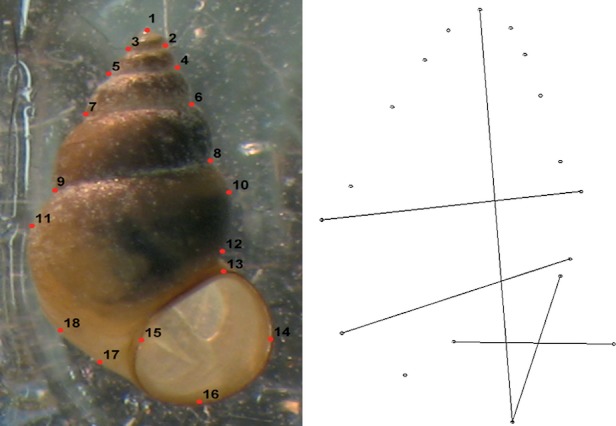
Eighteen landmarks used in morphometric analysis (left). Interlandmark distances used to calculate traditional length measurements on the shell (right). The following traditional length measurements were calculated: shell height, upper body whorl width, lower body whorl width, aperture width and aperture height.

Morphometric landmarks were chosen that are likely to present homologous points on the shell. Homologous points are defined by two criteria: distinctness from other locations and recognizable in all specimens (Zelditch et al. 2004). Thirteen homologous points were found on *P. antipodarum* (Fig. 1) including the apex (LM 1), whorls grooves (LM 2–9), and the aperture (LM 13–16). Body whorl landmark homology (LM 10–12, 18–17) was more problematic given the lack of basal cords and sharp narrow curves *P. antipodarum* often exhibited.

### Geometric morphometric analysis

A common method to analyze shape is geometric morphometrics. Morphometrics is a quantitative method of addressing shape comparisons using digitized landmark points (Zelditch et al. 2004). This process is more powerful than older methods of measuring height and width as it measures the overall shape of the entire organism.

The 18 landmark points were digitized from photos using TPSDig Version 2 (Rohlf 1997). Geometric morphometric analyses were conducted using these digitized landmarks. The file of digitized coordinates was opened in CoordGen6 (Sheets 2004), which was used to scale digitized landmarks to unit centroid size, and rotated to minimize the summed squared distances between homologous landmarks. This standard alignment known as Procrustes alignment removes size differences among specimens while retaining allometric relationships, making it possible to analyze shape independent of size (Zelditch et al. 2004). Thus, the effects of non-shape information (position, orientation, and scale) were mathematically eliminated from these landmark configurations using a generalized Procrustes analysis.

### Canonical variate analysis

A Canonical Variate Analysis (CVA) was conducted on the 18 digitized landmark points on *P. antipodarum* to determine the presence of morphological differences in shell shape between generations and among the three populations (Sheets 2004). A total of three separate CVAs were conducted: one for the parental generation, a second for the F_1_ generation, and a third containing both generations. A CVA mathematically optimizes between-group differences relative to within-group variation (Zelditch et al. 2004). In other words, a CVA emphasizes the differences that vary most between groups (populations or generations) while minimizing within-group variation, making it easier to discern which characteristics are unique to each group.

A CVA finds the axes that optimize between-group differences relative to within-group variation using partial warp scores. Partial warp scores are computed to a common reference, then a MANOVA is conducted followed by the CVA. This determines the number of distinct CV axes present in the data at *P* = 0.05 significance, and computes the canonical variate scores of all the specimens in the data set. To determine the number of significant CVs, Bartlett's test (1947) is employed to test for differences in Wilk's lambda (λ) value. Wilk's λ is the sum of squares within groups divided by the total sum of squares within and between groups:


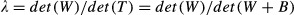


where det is the determinant of the matrix. Bartlett's test uses the following formula:


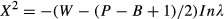


where *X*^*2*^ has an approximately chi-squared distribution, *W* is the degrees of freedom for the within-group sum of squares, *B* is the degrees of freedom for the between-group sum of squares, and *P* is the number of variables to determine if there are *G = B + 1* distinct groups. The degree of freedom within is *W = N − B*, where *N* is the total number of samples (Sheets 2004; Zelditch et al. 2004). The CVA also conducts a group assessment test in which specimens were assigned into groups based on their morphological variability. This assessment test is based on Mahalanobis distances, which are the distances in the space defined by the significant CV axes. All Canonical Variate Analyses were performed in CVAGen6j (Sheets 2004).

Mean landmark plots were also generated to visualize the general shape differences between the different groups. Mean landmark plots display the mean location of each of the 18 landmarks for each group analyzed in the CVA. All plots and grids were generated using PCAGen6 and CVAGen6j (Sheets 2004).

### Traditional length measurements analysis

Traditional length measurements were calculated using TmorphGen6 (Sheets 2004). This program generates a set of traditional length measurements from a geometric landmark data set of paired coordinate measurements. Unlike the canonical variate analysis, these calculations do not use the Procrustes alignment, so differences in sizes can be seen in these measurements. Potential problems were minimized by measuring offspring after they reached their full adult size. The following length measurements were calculated: shell height between landmarks 1 and 16, upper body whorl width between landmarks 10 and 11, lower body whorl width between landmarks 12 and 18, aperture width between landmarks 14 and 15, and lastly aperture height between landmarks 13 and 16 (Fig 1).

### Statistical analysis

A univariate ANOVA was used to compare morphological differences detected by the CVA among populations in the parental generation. A split-plot design with population, parental lineage nested within population, generation, and the generation*population interaction as factors was used to compare morphological differences detected by the CVA between the parental and the F_1_ generations. The offspring data were averaged out for each mother to account for unequal replication of offspring. This same design was applied when comparing traditional length measurements between the two generations. A bivariate ANOVA with parental lineages nested within populations was used to compare morphological differences detected by the CVA among populations of the F_1_ generation under a common environment (Proc GLM, Type III Sums of Squares, SAS Version 9.1 SAS Institute; Cary, North Carolina, USA).

If among-population differences in shell morphology are reduced in the F_1_ generation in a common garden, then the variation among wild-caught individuals from the three populations must be partially environmentally based plasticity. On the other hand, if among-population differences should persist in the F_1_ generation, then the among-population variation is predominantly genetically based. A significant effect of population in the F_1_ generation analysis would be consistent with an evolved genetically based response. In addition, shell morphology of parental and F_1_ snails should be the same if all the variation is genetically based, but should differ if it is partially environmentally plastic. A significant effect of generation would mean that shell morphology differs between the parental and F_1_ generations, suggesting a plastic response. A significant generation by population interaction would indicate a differential expression of evolved versus plastic variation in shell morphology across populations.

## Results

### Shell morphology variation in the parental generation

**Figure 2:**
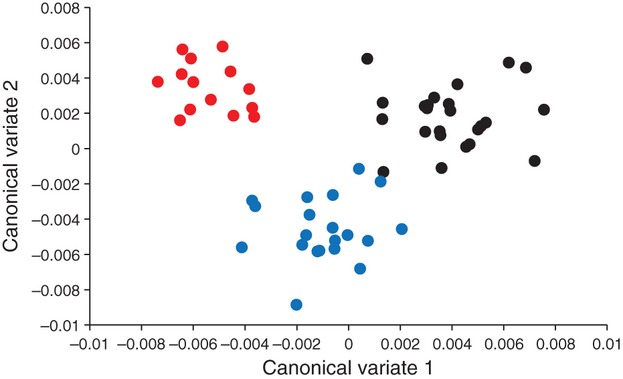
Canonical Variate Analysis plot of parental lineages. The parental generation was comprised of three distinct populations: Bear River, ID (black circles), Green River, UT (blue circles), and Polecat Creek, WY (red circles). Canonical Variate 1 was significant (*P* < 0.0001) and comprised 56.1% of the total variation. Canonical Variate 2 was also significant (*P* < 0.0001) and comprised 43.4% of the total variation.

The CVA conducted on the parental generation identified two significant canonical axes (Fig. 2). The assignment test grouped 98% of the snails to the correct population (Appendix A2). The ANOVA found significant differences among populations in overall shell morphology in the parental generation. The effect of population on shell morphology was significant along both CV1 (F_2,57_ = 116.83, *P* < 0.001) and CV2 (F_2,57_ = 101, *P* < 0.001). CV1 was mostly characterized by differentiation in the body whorl. A pair-wise comparison of CV1 means among populations found all to be significantly different from one another (*P* < 0.001). CV2 depicts differences in the aperture and the apex. A pair-wise comparison of CV2 means among populations found all to be significantly different (*P* < 0.001) except for Bear River and Polecat Creek (*P* = 0.052).

Pair-wise comparisons of the five traditional length measurements revealed significant differences among all populations in the parental generation (Appendices A3–A7). Green River snails exhibited the largest shell morphs followed by Bear River snails, while Polecat Creek snails exhibited the smallest shell morph in terms of the five traditional length measurements.

### Shell morphology differences between generations

**Figure 3:**
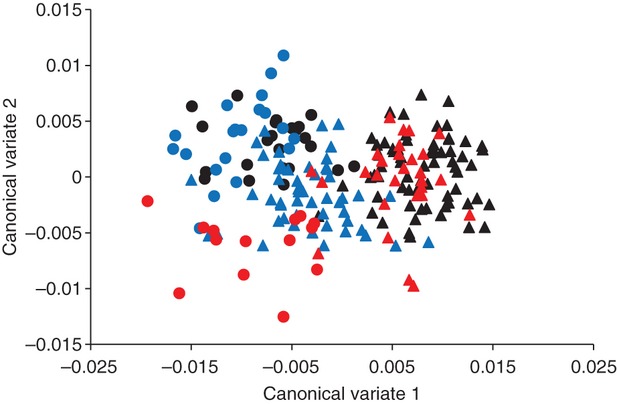
Canonical Variate Analysis plot of paternal and F_1_ generations. The parental generation was comprised of three distinct populations: Bear River, ID (black circles), Green River, UT (blue circles), and Polecat Creek, WY (red circles). The F_1_ generation was comprised of three distinct ancestral populations: Bear River, ID (black triangles), Green River, UT (blue triangles), and Polecat Creek, WY (red triangles). Canonical Variate 1 was significant (*P* < 0.0001) and comprised 61.7% of the total variation. Canonical Variate 2 was also significant (*P* < 0.0001) and comprised 11.9% of the total variation.

The CVA conducted on both parental and F_1_ generations identified two significant canonical axes (Fig. 3). The assignment test grouped 81% of the snails to the correct population (Table 1). The ANOVA showed that differences in overall shell morphology among the parental and F_1_ generations were significant (Table 2). The effect of generation was significant for CV1 (F_1,57_ = 314.80, *P* < 0.001), but not CV2 (F_1,57_ = 1.03, *P* = 0.325). CV1 was mostly characterized by differentiation in the body whorl and aperture. A pair-wise comparison of CV1 means between parental and F_1_ generations found significant differences among all populations (*P* < 0.001). CV2 displayed differences in the apex and the body whorl. A pair-wise comparison of CV2 means between parental and F_1_ lineages also found significant differences among all populations (*P* < 0.001).

**Table 1:** Group assignment from CVA-Mahalanobis distances of *P. antipodarum* parental and F_1_ lineages

Site	Bear River	Bear River F_1_	Green River	Green River F_1_	Polecat Creek	Polecat Creek F_1_
Bear River	21	0	1	2	0	0
Bear River F_1_	0	52	0	0	1	13
Green River	1	0	18	0	1	0
Green River F_1_	7	1	2	39	2	2
Polecat Creek	0	0	0	0	14	0
Polecat Creek F_1_	0	2	0	3	1	22

Original groups based on sites are placed along rows, while CVA groups based on morphological variability are placed along columns. The parental Bear River site consisted of 24 individuals, the parental Green River site consisted of 20 individuals, and the parental Polecat Creek site consisted of 14 individuals. The group assignment test placed 88% of Bear River parental snails, 90% of Green River parental snails, and 100% of Polecat Creek parental snails to the correct site and lineage. The Bear River F_1_ lineage consisted of 66 individuals, Green River F_1_ lineage consisted of 53 individuals, and Polecat Creek F_1_ lineage consisted of 28 individuals. The group assignment test placed 79% of F_1_ Bear River snails, 74% of F_1_ Green River snails, and 78% F_1_ Polecat Creek snails to the correct site. Only 19% of snails from both lineages were assigned to an incorrect site. The largest number of incorrectly assigned snails was 13 F_1_ Bear River snails being erroneously categorized as F_1_ Polecat Creek Snails.

**Table 2:** ANOVA results for the effects of Population, Generation, Parent(Population), and Population*Generation for CV1, CV2, and traditional length measurements

	Source	df	SS	MS	F	*P*-value
CV1	Population	2	0.00129179	0.00064590	52.38	<.0001
	Parent(Population)	57	0.00071685	0.00001303	1.06	0.4190
	Generation	1	0.00388179	0.00388179	314.80	<.0001
	Population*Generation	2	0.00044691	0.00022345	18.12	<.0001
	Error	57	0.00067820	0.00001233		
CV2	Population	2	0.00049401	0.00024700	28.38	<.0001
	Parent(Population)	57	0.00038994	0.00000709	0.81	0.7754
	Generation	1	0.00000894	0.00000894	1.03	0.3152
	Population*Generation	2	0.00046122	0.00023061	26.49	<.0001
	Error	57	0.00047875	0.00000870		
Shell height	Population	2	11.61244275	5.80622137	37.11	<.0001
	Parent(Population)	57	6.78707813	0.12340142	0.79	0.8094
	Generation	1	6.28066251	6.28066251	40.14	<.0001
	Population*Generation	2	3.19299183	1.59649592	10.20	0.0002
	Error	57	8.60625535	0.15647737		
Aperture width	Population	2	0.94797801	0.47398900	42.38	<.0001
	Parent(Population)	57	0.28761938	0.00522944	0.47	0.9972
	Generation	1	0.72694551	0.72694551	64.99	<.0001
	Population*Generation	2	0.26109001	0.13054500	11.67	<.0001
	Error	57	0.61519694	0.01118540		
Aperture height	Population	2	0.84293996	0.42146998	36.58	<.0001
	Parent(Population)	57	0.56082157	0.01019676	0.88	0.6740
	Generation	1	0.29803574	0.29803574	25.87	<.0001
	Population*Generation	2	0.60167300	0.30083650	26.11	<.0001
	Error	57	0.63370891	0.01152198		
Upper body whorl width	Population	2	2.23038632	1.11519316	50.42	<.0001
	Parent(Population)	57	0.85622523	0.01556773	0.70	0.9020
	Generation	1	0.90572389	0.90572389	40.95	<.0001
	Population*Generation	2	0.84636116	0.42318058	19.13	<.0001
	Error	57	1.21644729	0.02211722		
Lower body whorl width	Population	2	1.36487791	0.68243896	44.07	<.0001
	Parent(Population)	57	0.76428557	0.01389610	0.90	0.6552
	Generation	1	0.51271934	0.51271934	33.11	<.0001
	Population*Generation	2	0.81835942	0.40917971	26.42	<.0001
	Error	57	0.85166221	0.01548477		

The effect of parental lineages nested within populations is not significant for any shell shape traits suggesting that any maternal effects on F_1_ shell morphology are negligible. The F_1_ generation was smaller than their ancestral parental generation. However, significant among-population differences were maintained in the F_1_ lineages as indicated by the significant effect of population in all shell shape measurements.

There was a significant population by generation effect for CV1 (F_2,57_ = 18.12, *P* < 0.001) and CV2 (F_2,57_ = 26.49, *P* < 0.001), where the CV means for the F_1_ generation were significantly higher than those of the parental generation. The F_1_ generation exhibited parallel higher mean CV1 values than the parental generation. For CV2, the F_1_ generation exhibited lower CV2 means with the exception of Polecat Creek where the opposite trend was observed.

The effect of generation and population by generation interaction was significant for all five traditional length measurements (Table 2). Pair-wise comparisons of the five length measurements found some significant differences between parental and F_1_ generations for two of the three populations (Appendices A3–A7). Green River and Bear River offspring in the F_1_ generation were significantly different from their mothers in the parental generation for all five length measurements while the Polecat Creek offspring did not significantly differ from their mothers in any of the five length measurements (Appendices A3–A7). Green River and Bear River offspring in the F_1_ generation were shorter than the parental generation in all five traditional length measurements while Polecat Creek offspring in the F_1_ generation differed very little from their mothers in the parental generation in terms of overall length.

### Shell morphology differences among populations in the F_1_ generation

**Figure 4:**
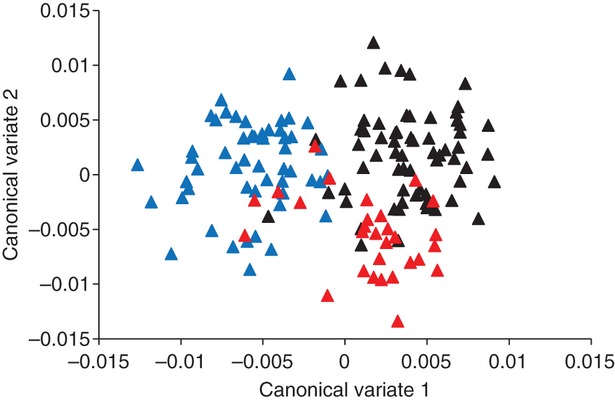
Canonical Variate Analysis plot of F_1_ lineages. The F_1_ generation was comprised of three distinct ancestral populations: Bear River, ID (black triangles), Green River, UT (blue triangles), and Polecat Creek, WY (red triangles). Canonical Variate 1 was significant (*P* < 0.0001) and comprised 84.5% of the total variation. Canonical Variate 2 was not significant (*P* = 0.15) and comprised 15.3% of the total variation.

The CVA conducted on the F_1_ generation identified one significant canonical axis (Fig. 4). The assignment test grouped 87% of the snails to the correct population (Appendix A8). The ANOVA showed that differences in overall shell morphology among populations in the F_1_ generation were significant. The effect of population on shell morphology was significant along both CV1 (F_2,90_ = 149.93, *P* < 0.001) and CV2 (F_2,90_ = 30.31, *P* < 0.001). The effect of parental lineage nested within population was not significant for either CV1 (F_57, 90_ = 0.63, *P* = 0.968) or CV2 (F_57,90_ = 1.01, *P* = 0.479), suggesting that any maternal effects on shell shape are negligible. CV1 represents differences in the body whorl and aperture. A pair-wise comparison of CV1 means found all populations to be significantly different (*P* < 0.001). CV2 represents mostly differentiation within the apex. A pair-wise comparison of CV2 means among populations found all populations to be significantly different (*P* < 0.001), although the Bear River and Green River was marginally non-significant (*P* = 0.064).

Pair-wise comparisons of the five traditional length measurements revealed that among-population differences were smaller in the F_1_ generation. For shell height, aperture width, and upper body whorl width, the only populations of the F_1_ generation that were significantly different from each other were Green River and Polecat Creek (Appendices A3–A7). There were no significant differences in aperture height and lower body whorl width among populations in the F_1_ generation (Appendices A3–A7). Like their parental-generation mothers, Green River snails were the largest, Bear River snails were intermediate, and Polecat Creek snails were the smallest in terms of shell height, aperture width, and upper body whorl width.

## Discussion

This common garden experiment sought to determine the importance of phenotypic plasticity and adaptive evolution in shell morphology of *P. antipodarum*. The generational CVA and traditional length measurement comparisons found morphological differences between the parental and F_1_ generations suggesting a plastic response. However, the F_1_ generation CVA and traditional length measurements indicate a genetic component. Both plasticity and evolution seem to be driving variation in shell morphology. The three parental populations exhibited shell morphs consistent with the water velocity of their corresponding environments while the F_1_ generation raised in a common lab environment exhibited shell morphs more suited to a low-flow environment. Although shell responses to a common environment appear to match predictions for optimal shell fitness (Vermeij 1995), it remains unclear whether or not these responses are truly adaptive. As *P. antipodarum* appear to exhibit morphological plasticity in their native range (Negovetic and Jokela 2001; Haase 2003; Holomuzki and Biggs 2006), invasive genotypes are predicted to exhibit some plasticity in shell shape as well. Differences between the parental and F_1_ generations as well as the attenuation of among-population differences in a common environment would indicate that variation among natural populations in shell shape was due to a plastic response. In fact, the F_1_ generation was significantly different from the parental generation in overall shape as indicated by the Canonical Variate Analysis. However, only Green River and Bear River F_1_ populations were significantly shorter in the five traditional length measurements than their ancestral mothers. While among-populations differences were reduced in F_1_ generation, the CVA revealed two significant canonical axes indicating that the offspring's overall shell shape retained some among-population differences. This trend was confirmed when traditional length measurements revealed among-population differences in shell size was retained, but overall shell size seemed to decrease in the F_1_ individuals. The reduction in among-population differences may be evidence of incomplete plasticity or an incomplete adaptive response to the new lab environment optimum (Ghalambor et al. 2007). Induced phenotypic changes in morphology may take more than one generation to occur and may even become canalized after the interaction is over (Agrawal 2001). Evidence for a plastic response would be stronger if among-populations differences continued to diminish over multiple generations and if the same trend was observed under multiple common garden environments (Crispo 2008).

Significant among-population differences in the F_1_ generation in a common garden suggest genetically based differences in shell shape responses to the environment. Shell shape variation in the F_1_ generation paralleled that of their ancestral lineages. Green River F_1_ population members exhibited the longest shell height, aperture width, and upper body whorl width while Polecat Creek F_1_ individuals exhibited the shortest lengths in these traits; this same pattern of variation was seen in the parental lineages. It is possible that some portion of these among-population differences in the F_1_ generation resulted from residual environmental effects that were not erased by rearing in a common lab environment (Crispo 2008). The portion that is genetically based might be surprising given that there is currently no evidence of genetic variation among these three populations (Dybdahl and Drown 2011). Other studies suggest that there is the potential for genetic variation among *P. antipodarum* populations in the Columbia and Snake Rivers (Dybdahl and Kane 2005; Hershler et al. 2010) suggesting the potential for adaptive evolution.

Although adaptive evolution and phenotypic plasticity have often been considered dichotomously as explanations for invasion success, recent mounting evidence argues that these two mechanisms are not mutually exclusive (Crispo 2008; Lande 2009). For example, a study of Arctic char morphological variation found significant between-generation differences (wild vs. lab-raised fish), suggesting a role for environmental modification in explaining patterns in natural populations (Adams and Huntingford 2004). Our results mirror these, because we also found significant differences in shell morphology among populations raised in a common environment, but differences between parental and F_1_ generations. At the same time, distinct morphs retained differences in a common garden, suggesting that some component of natural variation is genetically based. In addition, a significant population by generation effect suggests that each population responded differently to the lab environment. It remains to be demonstrated in this and other studies whether plasticity drives phenotypic change, followed by genetic changes in the direction of the plastic response (Fordyce 2006; Crispo 2008; Lande 2009).

Whether or not plasticity drives evolution, variation among populations is consistent with environmental responses in shell morphology. The shell morphs in the parental populations appear to reflect their natural habitat's water velocity. The fitness of coastal marine snails has been linked to shell morphs adapted to different levels of wave exposure (Struhsaker 1968; Janson and Sundberg 1983; Rolan-Alvarez et al. 1997; Denny and Blanchette 2000). In their native range, *P. antipodarum* have been shown to exhibit larger shell morphs in higher flow streams (Haase 2003). Larger and wider snail feet result in a greater attachment area that can withstand stronger currents (Dussart 1987) despite the increased effects of lift and drag forces associated with larger surface areas (Statzner and Holm 1989). Green River snails had the largest overall size in shell height, aperture height, aperture width, and body whorl width followed by Bear River. Both the Green River and Bear River sample populations were located downstream from dams suggesting that these populations may experience periods of high flow rates (Vanicek 1970). Bear River is subject to very strong currents in the summer (Drown, personal communication). On the other hand, Polecat Creek has low water velocity and its flow rates are relatively consistent throughout the year (Hall et al. 2003). Polecat Creek snails were the smallest in overall size, much like snails in the native range inhabiting low-flow sections of streams (Haase 2003).

The F_1_ generation's smaller shell morphs suggest a shift to a low-flow environment. Green River and Bear River F_1_ lineages experienced an overall decrease in size that can be attributed to the rearing environment, but some among-population differences were maintained. As in the parental populations, F_1_ Green River individuals were significantly longer and wider than the F_1_ Polecat Creek population. On the other hand, the Polecat Creek F_1_ lineage was not significantly different from their parental lineage in any of the traditional length measurements, but did differ in overall shell shape. This lack of change in shell traits associated with water flow may be due to the similarity in flow rate between Polecat Creek and the common environment.

In conclusion, the larger, high-flow shell morphs shifted to a smaller shell morph more suited to a low-flow environment within a single generation of lab rearing (Dussart 1987; Vermeij 1995). However, we cannot ascertain whether or not shell variation in the parental or F_1_ generations is adaptive. Shell morph and fitness measurements under multiple flow environments could ascertain whether or not *P. antipodarum* truly exhibit adaptive responses in shell morphology.

This common garden experiment suggests that both plasticity and evolution influence shell shape variation in invasive populations of *P. antipodarum*. Significant differences in shell size and shape between the parental and F_1_ generations suggest a plastic response, while among-population differences in the common garden environment in shell shape indicate a genetic component. Our findings on shell morphs are similar to studies of ecotypes across geographic ranges that attribute phenotypic differentiations to a combination of plastic and genetic differences (Kingsolver and Huey 1998; Gilchrist and Huey 2004; Chevin and Lande 2011). Because our common garden experiment lasted only a single generation, it is not clear what portion of the among-population differences were due to fixed genetic differences. The adaptive value of shell variation seems reasonable given the association between flow regime and shell morph (see also Kistner and Dybdahl, in revision). The role of plastic adaptation to each environment is supported by the shift by the larger shell morphs from high flow to a smaller shell morph more suited to a low-flow environment within a single lab generation. Further study is required to determine if *P*. *antipodarum*'s success across wide environmental gradients in the western United States over a short span of about two decades (Kerans et al. 2005; Hall et al. 2006) results from more than plastic variation in traits like shell shape.

## Appendix

**Appendix A1:** Collection information for *P. antipodarum* common garden populations

Collection Location	State, Nearest City	Latitude	Longitude	UMT Coordinates (X,Y, Zone)
Polecat Creek	WY, Flagg Ranch	44.1077°N	110.6836°W	525321, 4883884, 12
Bear River at Black Canyon	ID, Soda Springs	42.32580°N	111.47905°W	434596, 4709987, 12
Green River at Little Hole	UT, Manila	40.54721°N	109.18936°W	653319, 4490070, 12

**Appendix A2:** Group assignment from CVA-Mahalanobis distances of *P. antipodarum* paternal lineages

	Bear River	Green River	Polecat Creek
Bear River	23	1	0
Green River	0	20	0
Polecat Creek	0	0	14

Original groups based on populations are placed along rows, while CVA groups based on morphological variability are placed along columns. The paternal Bear River population consisted of 24 individuals, Green River consisted of 20 individuals, and Polecat Creek consisted of 14 individuals. The group assignment test placed 96% of Bear River snails, 100% of Green River snails, and 100% Polecat Creek snails to the correct population. Only one snail of the 58 maternal snails was assigned to an incorrect population.

**Appendix A3:** ANOVA results for shell height pair-wise comparisons among Generations (F_1_ for offspring) and Populations

Population	Bear River	Bear River F_1_	Green River	Green River F_1_	Polecat Creek	Polecat Creek F_1_
Bear River	–	<0.001	<0.001	0.005	<0.001	<0.001
Bear River F_1_	<0.001	–	<.0001	0.137	0.120	0.106
Green River	<0.001	<0.001	–	<.0001	<0.001	<0.001
Green River F_1_	0.005	0.137	<.0001	–	0.006	0.006
Polecat Creek	<0.001	0.120	<.0001	0.006	–	0.955
Polecat Creek F_1_	<0.001	0.106	<.0001	0.006	0.955	–

**Appendix A4:** ANOVA results for aperture width pair-wise comparisons among Generations (F_1_ for offspring) and Populations

Population	Bear River	Bear River F_1_	Green River	Green River F_1_	Polecat Creek	Polecat Creek F_1_
Bear River	–	<0.001	<0.001	0.007	<0.001	<0.001
Bear River F_1_	<0.001	–	<0.001	0.084	0.490	0.115
Green River M	<0.001	<.0001	–	<0.001	<0.001	<0.001
Green River F_1_	<0.001	0.084	<.0001	–	0.032	0.003
Polecat Creek	<0.001	0.490	<.0001	0.032	–	0.422
Polecat Creek F_1_	<0.001	0.115	<.0001	0.003	0.422	–

**Appendix A5:** ANOVA results for aperture height pair-wise comparisons among Generations (F_1_ for offspring) and Populations

Population	Bear River	Bear River F_1_	Green River	Green River F_1_	Polecat Creek	Polecat Creek F_1_
Bear River	–	<0.001	<0.001	<0.001	<.0001	<.0001
Bear River F_1_	<0.001	–	<0.001	0.525	<0.001	0.142
Green River	<0.001	<0.001	–	<.0001	<.0001	<.0001
Green River F_1_	<0.001	0.525	<.0001	–	0.002	0.381
Polecat Creek	<0.001	<0.001	<.0001	0.002	–	0.027
Polecat Creek F_1_	< 0.001	0.142	<.0001	0.381	0.027	–

**Appendix A6:** ANOVA results for upper body whorl width (mm) pair-wise comparisons among Generations (F_1_ for offspring) and Populations

Population	Bear River	Bear River F_1_	Green River	Green River F_1_	Polecat Creek	Polecat Creek F_1_
Bear River	–	<0.001	<0.001	<0.001	<0.001	<0.001
Bear River F_1_	<0.001	–	<0.001	0.354	0.002	0.055
Green River	<0.001	<.0001	–	<0.001	<0.001	<0.001
Green River F_1_	<0.001	0.354	<0.001	–	<0.001	0.009
Polecat Creek	<0.001	0.002	<0.001	<0.001	–	0.248
Polecat Creek F_1_	<0.001	0.055	<0.001	0.009	0.248	–

**Appendix A7:** ANOVA results for lower body whorl width (mm) pair-wise comparisons between Generation (F_1_ for offspring) and Populations

Population	Bear River	Bear River F_1_	Green River	Green River F_1_	Polecat Creek	Polecat Creek F_1_
Bear River	–	<0.001	<0.001	<0.001	<0.001	<0.001
Bear River F_1_	<0.001	–	<0.001	0.879	<0.001	0.113
Green River	<0.001	<0.001	–	<0.001	<0.001	<0.001
Green River F_1_	<0.001	0.879	<0.001	–	<0.001	0.161
Polecat Creek	<0.001	<0.001	<0.001	<0.001	–	0.036
Polecat Creek F_1_	<0.001	0.113	<0.001	0.161	0.036	–

**Appendix A8:** Group assignment from CVA-Mahalanobis distances of *P. antipodarum* F_1_ lineages

	Bear River	Green River	Polecat Creek
Bear River	55	2	9
Green River	0	51	2
Polecat Creek	2	4	22

Original groups based on populations are placed along rows, while CVA groups based on morphological variability are placed along columns. The Bear River F_1_ population consisted of 66 individuals, Green River consisted of 53 individuals, and Polecat Creek consisted of 28 individuals. The group assignment test placed 83% of Bear River snails, 96% of Green River snails, and 78% Polecat Creek snails to the correct population. Only 13% of F_1_ snails were assigned to an incorrect population.
